# London Medico-Chirurgical Review

**Published:** 1845-11-01

**Authors:** 


*London Medico-Chirurgical Review.*—We would refer our readers to the advertisement of this periodical in our advertising sheet. It has been so long and favorably known to the medical public, that it would be supererogation in us to bestow commendations upon it. It possesses one feature which renders it invaluable to those whose circumstances do not enable them to make extensive bibliographical pnrchases. It contains analytical reviews of all the prominent publications as they appear, presenting their distinguishing characteristics in a condensed form, thereby economising both the time and money of the reader, and being readily available for after reference. A complete set of this periodical for the 20 or 30 years it has been in existence is almost as valuable as a library containing all the current medical literature of the same period.

